# Expression profiling of prostate cancer tissue delineates genes associated with recurrence after prostatectomy

**DOI:** 10.1038/srep16018

**Published:** 2015-11-02

**Authors:** Martin Mørck Mortensen, Søren Høyer, Anne-Sophie Lynnerup, Torben Falck Ørntoft, Karina Dalsgaard Sørensen, Michael Borre, Lars Dyrskjøt

**Affiliations:** 1 Department of Molecular Medicine, Aarhus University Hospital, Brendstrupgårdsvej 100, DK-8200 Aarhus N, Denmark; 2 Department of Urology, Aarhus University Hospital, Brendstrupgårdsvej 100, DK-8200 Aarhus N, Denmark; 3 Department of Pathology, Aarhus University Hospital, Nørrebrogade 44, DK-8000 Aarhus C, Denmark

## Abstract

Prostate cancer is a leading cause of cancer death amongst males. The main clinical dilemma in treating prostate cancer is the high number of indolent cases that confer a significant risk of overtreatment. In this study, we have performed gene expression profiling of tumor tissue specimens from 36 patients with prostate cancer to identify transcripts that delineate aggressive and indolent cancer. Key genes were validated using previously published data and by tissue microarray analysis. Two molecular subgroups were identified with a significant overrepresentation of tumors from patients with biochemical recurrence in one of the groups. We successfully validated key transcripts association with recurrence using two publically available datasets totaling 669 patients. Twelve genes were found to be independent predictors of recurrence in multivariate logistical regression analysis. SFRP4 gene expression was consistently up regulated in patients with recurrence in all three datasets. Using an independent cohort of 536 prostate cancer patients we showed SFRP4 expression to be an independent predictor of recurrence after prostatectomy (HR = 1.35; p = 0.009). We identified SFRP4 to be associated with disease recurrence. Prospective studies are needed in order to assess the clinical usefulness of the identified key markers in this study.

Prostate cancer is the most prevalent cancer type in men in the western world^1^. As with many different cancer types curative treatment is possible in the case of localized disease, in which surgery or radiation therapy can be offered to the patient. Early diagnosis is therefore essential if the patient is to be cured from the disease. A distinct feature of prostate cancer is the high prevalence of indolent cancer lesions that have a long latency period before, if ever, causing morbidity or mortality for the patients. Historical data based on autopsy findings shows a prevalence of prostate cancer of 29% for males aged between 50 and 59 years increasing to all males aged >90 years^2^,^3^. In a study on prostate cancer patients not receiving curative treatment, Albertsen *et al.* showed that the risk of dying of their disease was 4%–7% within 15 years for low risk patients^4^. This high prevalence of indolent cancers confers a significant risk over diagnosis and over treatment.

Selecting patients with prostate cancer for therapy remains a major clinical dilemma in treating clinically localized prostate cancer. Screening tends to induce a stage shift towards lower tumor burden and more favorable prognosis especially in prostate cancer, where the rate of indolent cancer is very high. Finding better prognostic markers is therefore essential for better treatment strategies and for making screening for prostate cancer a more viable option. The utilization of full-genome expression microarrays is a potent tool for identifying novel biomarkers of progression in prostate cancer. Previous studies have shown that the method can identify gene transcripts that are differentially expressed and several gene expression signatures have been reported for predicting disease recurrence after surgery^5^,^6^,^7^,^8^,^9^,^10^,^11^. Other studies have applied expression microarrays to discover transcripts associated with systemic progression after surgery^12^,^13^ thus exploring a different clinical end-point for aggressive prostate cancer. Single transcripts associated with recurrence have been identified from expression microarray experiments, like PIM1 and HPN^14^, TRPM8-p8^15^, and MUC1 and AZGP1^7^ showing that expression microarray can also be used to detect gene candidates for other applications such as immunohistochemistry or q-RT-PCR detection.

Here, we have performed whole genome expression profiling of laser micro dissected tissue from 36 cancer samples and 14 normal prostate samples to delineate novel biomarkers of aggressive disease. Using unsupervised consensus cluster analysis, we identified a molecular subgroup of prostate cancer associated with disease aggressiveness. Furthermore, we identified gene clusters that were associated with the aggressive molecular subgroup, gene clusters that were found to be associated with invasive properties of the tumor, as well as cell cycle and mitosis when tested using Ingenuity Pathway Analysis (IPA) software. We successfully validated our key candidate markers in two independent patient cohorts at the transcript level, and further successfully validated the expression of SFRP4 at the protein level using a fourth independent cohort of 536 prostate cancer patients.

## Results

A total of 36 prostate cancers and 14 normal prostate samples were laser micro dissected and gene expression profiled using Affymetrix U133 2.0 Plus microarrays for delineation of molecular markers of disease aggressiveness. Clinical and histopathological data for all patients included in the study are listed in (Table 1). Overall, patients were followed for a median of 66 months (range 31–80 months) and 60% experienced disease recurrence.

### Analysis based on clinical outcome

Initially, we filtered the data to include only transcripts that showed a variance >1 across all samples. Hence, non-expressed or non-varying transcripts were excluded from analysis - in total 5732, probe-sets were included in the analysis following filtering. We delineated molecular markers by direct comparison between recurrent and non-recurrent cases. Only 11 probe-sets showed significant association with the risk of having recurrent disease (Bonferroni corrected for multiple testing), including 6 annotated genes (Table 2).

### Analysis of molecular subgroups

**Figure 1:**
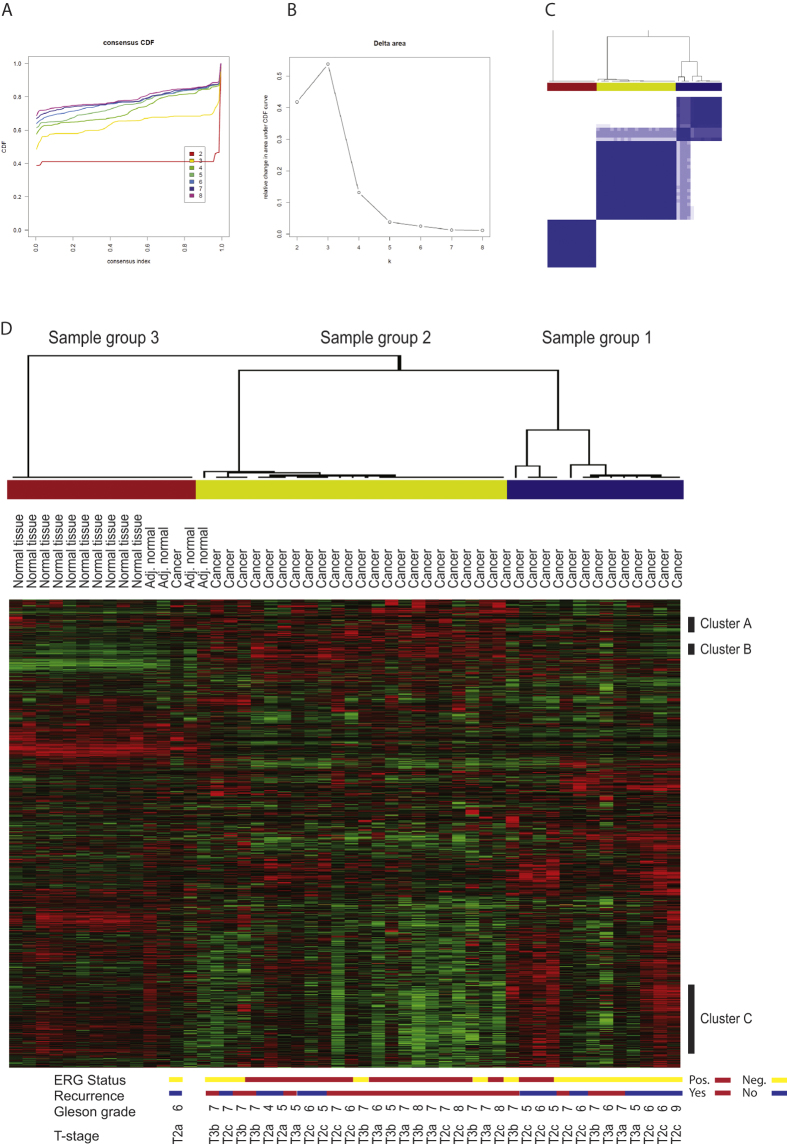
Consensus based cluster analysis of gene expression in prostate cancer and normal prostate. (**A**) plot of the cumulative distribution function (CDF) for each number of clusters tested. (**B**) Plot of changes in area under CDF curve; change from two to three groups (k = 3) gives the highest relative change in CDF, indicating that the data is best represented by three groups. (**C**) Consensus matrix using a three group model (k = 3). (**D**) Sorting of samples according to consensus cluster; red denotes up regulation of a gene, and green denotes down regulation, black is the median expression of the gene. Black bars to the right of the heat map show the selected key gene clusters colored bars represent the three sample clusters. T-stage, Gleason grade, recurrence status and ERG status is listed below the heat map.

We performed a consensus based (ConsensusClusterPlus) cluster analysis of the filtered dataset using 1000 re-samplings of the data (95% of samples retained in each re-sampling) to discover molecular subgroups associated with aggressiveness of the disease. The consensus based cluster analysis revealed three stable groups in the data (Fig. 1); group 1 with an over representation of non-recurrent cases, group 2 contained primarily recurrent cases and group 3 contained normal prostate samples and one cancer sample (Table 3, Fig. 1). There was a significant overrepresentation of recurrent cases in group 2 (named: aggressive molecular subgroup) compared to group 1 (named: indolent molecular subgroup) (p = 0.022, chi^2^ test). Sample RIN score did not correlate with the determined molecular subgroup (r = 0.22, p = 0.18).

**Figure 2:**
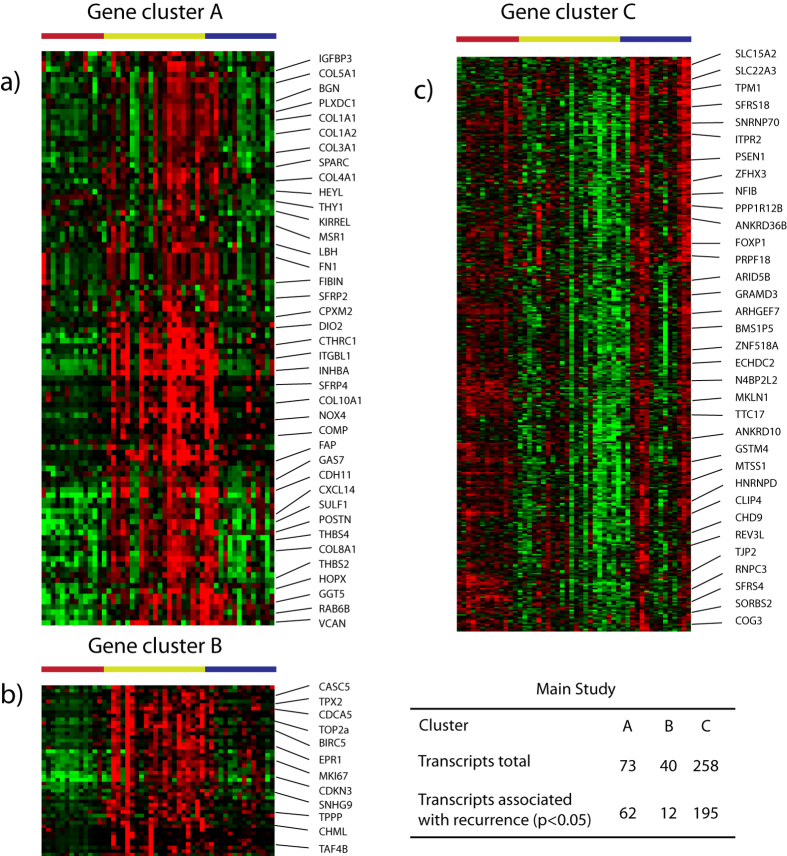
Selected gene clusters containing genes differentially expressed between the sample cluster groups. Top ranked transcripts associated with recurrence (p < 0.05, T-test) are shown for each cluster. (**a**) Invasive cluster A. (**b**) Cell cycle cluster B. (**c**) Tumor suppressors cluster C. (**d**) Overview of the total number of transcripts in each cluster and how many, that are associated with recurrence after surgery.

Several distinct clusters of genes were identified based on the expression profile according to the three sample clusters. Key clusters and genes are highlighted in Fig. 2. Molecular subgroups associated with aggressive disease have previously been identified by Markert *et al.*^16^, who tested 15 different signatures and identified five molecular subgroups. One subgroup was characterized by being enriched with the signatures ESC, PTEN-, p53-, MYC+ and proliferation and had the poorest overall survival. The subgroup with the second worst overall survival was characterized by harboring the TMPRSS2:ERG fusion. By using GSEA, we applied these previously identified signatures to our dataset using all patients and using recurrence status as discriminating parameter. None of the signatures were found to be significantly enriched (data not shown), but when testing the same signatures in relation to sample cluster groups 1 and 2, we found that the signatures ESC, proliferation, and ERG were significantly enriched in the aggressive molecular subgroup (p = 0.029 FDR = 0.20, p = 0.034 FDR = 0.23, p = 0.039 FDR = 0.21). For complete list, see Supplementary Table S1. The importance of the identified molecular subgroups was also emphasized by analyzing ERG expression. Expression of ERG mRNA is highly concordant with TMPRSS:ERG fusion status^17^. We found a distinct on/off expression with samples either expressing ERG at the baseline corresponding to no expression or expressing ERG at high levels. ERG expression was seen in 58% of the cancer samples. With ERG expression dichotomized in over and under the median we compared ERG expression status directly with recurrence as end-point, and we observed no significant association (p = 0.8; univariate cox regression). However, high ERG expression was significantly over represented in our aggressive molecular subgroup of patients (p = 0.002, chi^2^ test).

### Analysis of gene clusters

We identified several distinct clusters of genes that showed differential expression between the sample groups (see Figs 1 and 2). Clusters A, B and C included genes differentially expressed between group 2 (aggressive subgroup) and group 1 (indolent subgroup). Clusters A and B contained genes highly expressed in the aggressive molecular subgroup, whereas cluster C contained genes down regulated in the aggressive molecular subgroup. For each transcript in the gene clusters the direct association between expression level and recurrence status was calculated and the results are summarized in Fig. 2 (the full list of transcripts from each cluster is included in the Supplementary Tables S2–4). In order to determine the possible biological function of the transcript clusters we applied Ingenuity Pathway Analysis (IPA) to all genes in each cluster. Cluster A was enriched for cellular movement (p = 2.5 E-6) and growth and proliferation (1.06 E-06) pathways. Cluster B was highly enriched for genes involved in cell cycle regulation (p = 1.04 E-13), and DNA repair, replication, and recombination (p = 5.09 E-8). IPA thus indicated that the two clusters of genes are associated with different biological functions. Cluster C was enriched for a variety of rather unrelated molecular and cellular functions, including cellular assembly and organization (p = 3.5 E-4) and gene expression (p = 7.2 E-4). None of the clusters were enriched for transcripts associated with androgen receptor transcript factor activity.

### Validation in independent samples

In order to validate the identified molecular subgroups of prostate cancer associated with different outcomes, we used two publicly available expression datasets –Taylor *et al.* (2011) and Nakagawa *et al.* (2008)^13^,^18^. The detailed clinical data of the two validation cohorts are listed in (Table 1). First, the genes in each transcript cluster A, B and C were applied to the validation datasets using GSEA. Cluster A, containing transcripts involved in tumor invasion, was found to be significantly enriched in the patients with recurrent disease (p = 0.025, FDR = 0.02) in the Taylor dataset. Cluster C, containing genes down regulated in the aggressive molecular subgroup, was found to be significantly enriched in the patients having non-recurrent disease (p  < 0.001, FDR = 0.007), thus validating the association with recurrence as well as confirming the direction of association. A trend was observed for Cluster B, but the association was not significant (p = 0.094, FDR = 0.207) (Supplementary Table S5). For the Nakagawa dataset, no signatures reached significance but as the dataset only includes 1028 transcripts, the number of applicable genes from each cluster was reduced accordingly.

We then calculated the association of each individual transcript from the gene clusters with recurrence status in our two validation datasets using t-test. Only transcripts significantly associated with outcome in the main study were included in the validation. In the Taylor dataset, 36 of the 62 significant transcripts in cluster A were successfully validated (p < 0.05, students t-test), with the highest-ranking transcripts being INHBA and SFRP4. For cluster B five transcripts were successfully validated out of the 10, that were significantly associated with recurrent disease (p < 0.05), top ranked transcripts being TOP2A and CASC5. In cluster C, 69 of the 195 transcripts were successfully validated (p < 0.05, students t-test) with transcripts of the TPJ2 and TPM1 genes being highest ranked. The Nakagawa data set only contained probes for measuring 1028 gene transcripts; 18 transcripts from cluster A, 4 from cluster B and 6 transcripts from cluster C were included in the Nakagawa validation dataset and were significantly associated with recurrence in the main study. Overall 20 out of 28 transcripts from all three clusters were successfully validated in the Nakagawa data set (Supplementary Table S6).

Using a multivariate Cox regression model including Gleason score, t-stage, and preoperative PSA as well as the expression level of the gene, we found that 12 of the markers were independently associated with the risk of recurrence after prostatectomy (Supplementary Table S7) in the Nakagawa validation data set (p < 0.05). Highest ranking transcripts were INHBA, COL1A2, IGFBP3, MSR1 and SFRP4. The Taylor dataset included too few events in order to perform multivariate analysis.

### Tissue microarray validation of SFRP4

**Figure 3:**
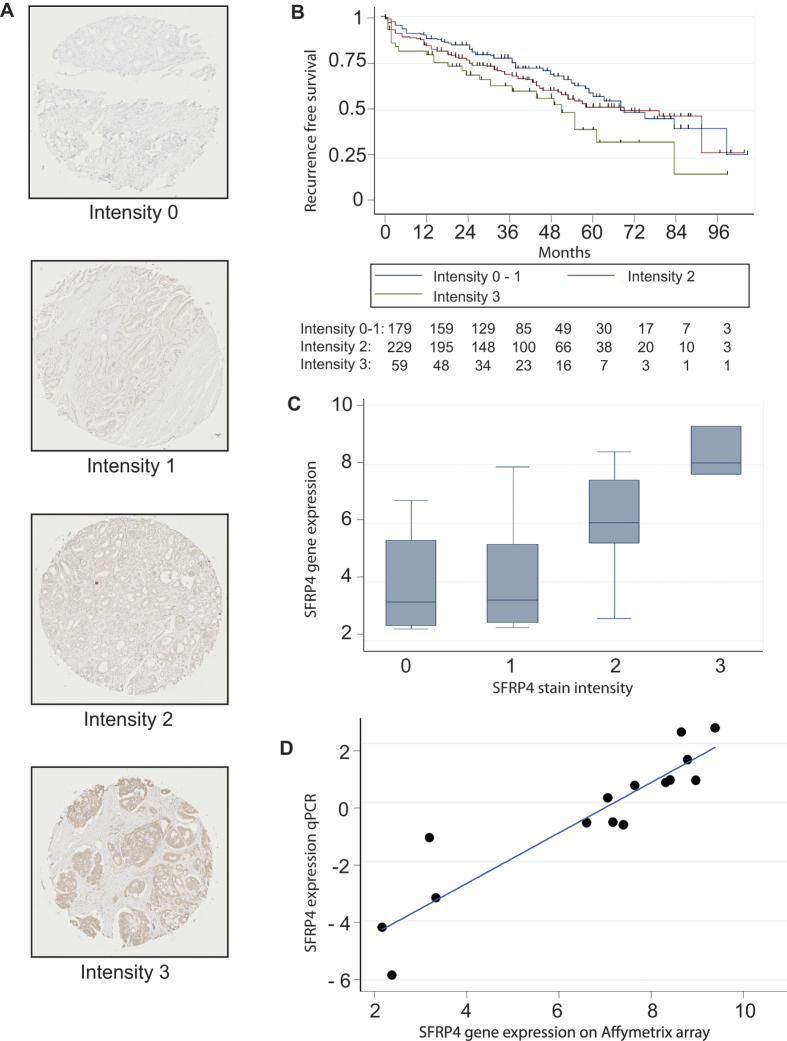
Expression of SFRP4 and correlation to outcome. (**A**) Example of each of the staining intensities 0-to 3 (**B**) Kaplan-Meier plot of recurrence free survival as a function of SFRP4 protein expression. (**C**) Correlation plot between the intensity of SFRP4 measured by IHC and the expression level of SFRP4 measured by microarray analysis from the same patients. (**D**) Correlation plot between SFRP4 gene expression measured by Affymetrix array and by q-RTPCR.

We used a TMA for validation of SFRP4 at the protein level. A summary of the clinical characteristics for all patients is shown in Table 1. Overall, 470 (90.9%) patients had one or more cores available for evaluation. Of these 470 patients 291 (52%) were scored similarly on all available cores, where cores differed in intensity from the same patient the highest score was used. Associations between clinical data and SFRP4 staining are shown in Supplementary Table S8. Kaplan-Meier survival curves showed that the risk of biochemical recurrence after prostatectomy increased with increased protein level of SFRP4 (log-rank test, p =  0.01 when comparing low and high intensity) (Fig. 3). Univariate Cox regression analysis showed a significant association between staining intensity and recurrence (HR = 1.30; p = 0.023), and when correcting for Gleason score, pathological t-stage, margin status, and preoperative PSA in multivariate analysis the association remained significant (HR = 1.35; p = 0.009) (Supplementary Table S9). Consequently, a high SFRP4 level was found to be an independent predictor of biochemical recurrence after prostatectomy. A previous study by Horvath *et al.* found that membranous SFRP4 staining was associated with improved outcome after RP^19^. However, no significant association between membranous SFRP4 staining and biochemical recurrence was seen in our patient set (data not shown).

Finally, 26 of the 36 patients included in the microarray study were also represented on the TMA, but were not included in the validation cohort. Comparison of the protein level and the gene expression data (Fig. 3) showed a positive correlation of 0.58 (Pearson). The specificity of the SFRP4 antibody was evaluated on western blotting which showed a band at 48 kDa, when tested on protein extracts from PC3 and DU145 prostate cancer cell lines, the predicted molecular weight of SFRP4 being in the range 43-55 kDa. In additions, we also verified the microarray probe specificity by performing q-RT-PCR using samples analyzed on the microarray platform (Fig. 3). Here we also found a positive correlation of 0.92 (Pearson).

## Discussion

The objective of our study was to utilize expression array analysis to delineate markers for disease recurrence after prostatectomy and to obtain knowledge about biological pathways associated with disease aggressiveness. Using consensus based unsupervised clustering of the expression data we found three stable groups in the data, and identified one molecular subgroup of prostate cancer associated with disease aggressiveness. Following the establishment of the molecular subgroups, we found three clusters of genes differentially expressed between the aggressive and the indolent subgroups. Two of the gene clusters were found to be representing two different functional groups of genes one containing genes involved in invasive properties, the other in cell cycle related functions. Substantial validation was performed; firstly, the gene clusters were validated successfully in the Taylor dataset, secondly the majority of the individual transcripts in the clusters were validated in the Taylor and Nakagawa datasets, and showed that 12 genes were independent predictors of recurrence highest ranked transcripts being INHBA, COL1A2, IGFBP3, MSR1 and SFRP4. Finally, the candidate gene SFRP4 was found to be an independent predictor of recurrence after prostatectomy tested on a tissue microarray in an independent patient cohort.

The existence of molecular subgroups in prostate cancer is an intuitive thought since there is a great difference in the behavior of the different prostate cancer cases. Previous expression profiling studies have shown that clustering algorithms performed on expression dataset can delineate subgroups of patients and that the established groups can have different clinical outcomes^7^,^16^. The aggressive subgroup established in our study was shown to correlate with the two subgroups with the worst prognosis in the Markert *et al.* study. The fact that both the ESC, proliferation and the ERG signatures were enriched in our aggressive molecular subgroup indicates that the aggressive subgroup in our study is composed of both groups from the Markert *et al.* study. The proportion of patients belonging to the aggressive subgroup is higher in our study (62%) than in the Markert study (29%). This could relate to differences in the patient cohorts being examined. Our study is comprised of patients selected for radical surgery, all patients were diagnosed on needle biopsy, and the patient cohort used in the Markert study comes from the study by Sboner^20^ where all patients were diagnosed from tissue taken during a TUR-P procedure - performed because of lower urinary tract symptoms. Thus, the cohorts are not comparable in terms of how patients were included, and the fact that the same molecular subgroups can actually be detected in this setting makes the finding even stronger. Further, we do not know whether the genes driving the patient sub groups are expressed in a sub group of the cells in the tumor or are common to all cells in the tumor. For SFRP4 there was some heterogenic expression with staining intensities differing between cores from the same patient in about 50% of the patients, whether this is true for the other transcripts remains to be investigated.

To determine the clinical usefulness of assessing SFRP4 expression further studies are needed, for instance studies testing the SFRP4 expression in an active surveillance patient cohort, or in relation to selecting patients for adjuvant endocrine therapy after radiotherapy.

Clinical use of array analysis to establish which molecular subgroup a given prostate cancer patient belongs to may be hindered by the expense of the test as well as the quality and quantity of RNA necessary to perform the array analysis with sufficient reproducibility. Finding individual transcripts or a short list of transcripts that can be assayed with immunohistochemistry or qPCR will be considerably more applicable in clinical use. The gene clusters identified in our study contain genes that are differentially expressed between molecular subgroups associated with biochemical recurrence, and thus contain transcripts that can potentially be used to identify patients with aggressive prostate cancer. The association with biochemical recurrence of the individual transcripts in the three gene clusters has been validated in two independent cohorts. The validation data is generated in geographically different cohorts of patients from North America as opposed to a European cohort in the main study. Tissue used in the Nakagawa dataset was formalin fixed paraffin embedded (FFPE), while in this study and in the Taylor study fresh frozen tissue biopsies were used. Finally, the array platform is different using a DASL platform in the Nakagawa study, exon array in the Taylor study and an Affymetrix platform in our study. The composition of the cohort in terms of clinical data was also different between the validation cohorts. The Taylor data set consists primarily of patients with a relatively low risk clinical profile and the Nakagawa data set contains a considerably larger proportion of high-risk patients with high Gleason score and advanced t-stage. Despite these differences, it was still possible to validate 40% of all the transcripts in the three gene clusters in the Taylor data set and additionally validate 20 of 28 possible transcripts in the Nakagawa data set, thus indicating that the association of the transcripts with recurrence was both reproducible and universal.

The SFRP4 gene is one of 5 known secreted frizzled proteins that function as WNT antagonists^21^. Altered SFRP4 expression leads to altered activation of the WNT pathway. Since WNT signaling promotes tumor growth, WNT antagonists generally function as tumor suppressors^22^. This is also the case for SFRP4, as several studies in different cancer types have shown that reduced levels of SFRP4 leads to a worse prognosis^23^,^24^,^25^. In prostate cancer increased SFRP4 expression have been shown to decrease proliferation in PC3 a prostate cancer cell line^19^, which is in agreement with the general theory regarding how WNT signaling works in cancer^22^. However, in other studies the opposite association has been shown, which was high SFRP4 expression was associated with poor prognosis^26^,^27^,^28^, so the role of SFRP4 in cancer is complex.

A unique trait of prostate cancer is the propensity to metastasize to bone. WNT signaling is well studied in bone and several studies confirm that inhibition of WNT signaling leads to inhibition of osteoblast function^29^. In this setting, SFRP4 has directly been shown to be able to mediate this osteoblast inhibition and thus lead to reduced bone mass^30^,^31^. The WNT mediated osteoblast inhibition has been shown to be implicated in bone metastasis from multiple myeloma patients where high levels of Dkk-1 a known WNT antagonist leads to impaired osteoblast function and lytic bone lesions^32^. In prostate cancer, Hall *et al.* showed that WNT inhibition with Dkk-1 in prostate cancer cell lines induced reduced osteoblast differentiation and a shift towards lytic lesions^33^. Although SFRP4 has not directly been shown to induce lytic bone metastasis like Dkk-1, it has been shown to inhibit osteoblast function in the same manner, so it can be speculated that SFRP4 can cause prostate cancer progression to metastatic disease through an increased ability to create bone lesions. The paradoxical growth retardation seen in previous studies of cancer cell lines might be outweighed by an increased ability to metastasize to bone, which can explain why high SFRP4 expression is associated with recurrence after prostatectomy.

The other top candidate INHBA from cluster D has been implicated in the formation of bone metastasis. INHBA forms a homodimer to create the physiological active substance Activin A, which is part of the TGF-Beta superfamily^34^. Circulating levels of Activin A have been associated with bone metastasis in prostate and breast cancer as well as in myeloma patients^35^,^36^,^37^ and in a mouse model testing development of bone metastasis with myeloma derived cells, blocking Activin A directly inhibited tumor growth in bone and prevented bone absorption^38^. Activin A antagonists are currently being tested therapeutically treating bone metastasis and cancer related anemia^39^.

Predicting the risk of recurrence after curative treatment can have a great impact of the management of prostate cancer. The benefit of adjuvant radiation therapy following radical prostatectomy has been proven for patients with adverse disease characteristics. However not all patients in this group recur after prostatectomy and some patients with favorable characteristics also recur, which make patient selecting difficult. Currently three commercial gene expression signatures for disease aggressiveness are available (Prolaris^40^, OncotypeDx^41^ and Decipher^42^). There is an overlap between the transcripts identified in this study and the transcripts included in the commercially available signatures. E.g. the Prolaris signature has an eight gene overlap with the cell cycle cluster identified in this manuscript, the OncotypeDx signature has a four gene overlap including SFRP4, and the Decipher has an overlap of four genes. Although an association with recurrence is established for the gene signature identified here, the use of the identified genes to stratify patients to adjuvant radiation therapy will require further prospective studies. Furthermore, although we see overlap with previously published signatures of aggressiveness, the prognostic power of the here reported gene signature compared to other signatures still needs to be determined in independent studies.

## Methods

### Ethics statement

The study was approved by the Central Denmark Region Committees on Biomedical Research Ethics case number 2002-41-2640. Informed written consent was obtained from all patients. The study was carried out in accordance with the approved guidelines.

### Clinical samples

Samples for this study were provided by the Aarhus prostate cancer project consisting of all patients undergoing radical prostatectomy at the Dept. of Urology Aarhus University Hospital from 1995 to present. Clinical data were collected prospectively and recurrence status for all patients in the study was updated prior to inclusion in the study. The prostatectomy specimens were examined by a trained pathologist, pathological stage was assessed, and the Gleason grade of the tumor was determined. Serum PSA was measured prior to surgery by automated immunoassay using DPC Total PSA Immulite and expressed in ng/ml. Follow up after surgery has been conducted by PSA measurements at 3, 6 and 12 month postoperatively and thereafter biannually. Subsequent biochemical failure was defined as a PSA > 0.2 ng/ml. Biopsies were taken from the surgical specimen and immediately snap frozen. Normal samples were procured from patients with bladder cancer undergoing cystectomy and prostate biopsies were taken from the cystoprostatectomy specimen.

### Laser micro dissection and RNA extraction

Survey slides of the biopsies were evaluated by a trained uro-pathologist to ensure that only tumor tissue was included. Tumor tissue was taken from only one biopsy since often only one biopsy contained tumor tissue. Subsequently, slides were stained with cresyl-violet 1%, and the carcinoma cells were laser micro dissected using the PALM laser microbeam system. RNA extraction was performed using RNeasy Micro kit from Qiagen (Germany). The kit is specially optimized for extracting RNA from laser micro dissected tissue. The quantity and quality of the RNA extracted was measured using the Agilent 2100 Bioanalyzer with RNA Pico Chips from Agilent Technologies (Germany). A minimum of 7 ng of total RNA was extracted from each sample with a median RIN value of 5.9 (range 3.9–9.7). Correlations between the sample RIN score and clinical covariates have been calculated, and no significant correlation was found (data not shown). Similarly, the benign prostate tissue samples were laser micro dissected for procuring normal prostate epithelial cells.

### Microarray analysis

Total RNA was amplified and converted to cDNA using Nugen Pico-RNA system. The two-round amplification kit is optimized to amplify low volumes and poor quality RNA for Affymetrix array analysis. After amplification, the cDNA was fragmented and labeled using NuGen FL-Ovation kit, and loaded onto the Affymetrix U133 Plus 2.0 Gene Chip according to the manufacturer’s protocol. Each Gene Chip was scanned using the Affymetrix 3000 7 G Scanner. Data was RMA normalized and intensity measures generated using GeneSpringer version 11 software. The gene annotation file used was downloaded from the Affymetrix homepage and was HG-U133a plus 2.0 release 27. The complete data set has been made publically available through the Geo repository. The Geo accession number is GSE46602. Microarray data was filtered before analysis to avoid inclusion of non-varying transcripts. Only transcripts that showed an overall variance greater than 1 across all samples were included. The filtering criterion of variance greater than 1 reduced noise introduced by transcripts with minimal variation across samples. The filtering resulted in selection of roughly 10% of the transcripts. Class discovery was performed using ConsensusCluster Plus^43^ using 1000 re-samplings. Hierarchical cluster analysis of gene expression was performed using Cluster 3.0 software and gene expression was visualized using Java tree-view software^44^. Gene set enrichment analysis (GSEA) was performed using GSEA v2.07 software. Significant enrichment was accepted when the normalized p-value was below 0.05 and the false discovery rate was below 0.25, thus using the default significance levels^45^. Transcripts were matched across the validation data sets by gene symbol.

### qRT-PCR

Technical validation of the microarray result was performed for SFRP4 by q-RT-PCR. Total RNA from 15 patients was converted to cDNA. Sub sequentially amplified using Nugen Pico-RNA system. A Taq-man primer set targeting SFRP4 (assay id Hs00180066 m1*) was used for the real-time PCR and UBC was included for normalization, and measured using primers 5′-GATTTGGGTCGCGGTTCTT-3′ plus 5′-TGCCTTGACATTCTCGATGGT-3′ and SYBR Green PCR Master Mix (Applied Biosystems)^46^.

### Immunohistochemistry

A tissue microarray (TMA) was constructed containing 1 mm cores in triplicates from 517 patients undergoing radical prostatectomy. Tissue was taken from archival FFPE blocks, the Gleason grade was reassessed for all tumors, and full clinical follow-up concerning recurrence status was done. Immunohistochemical staining of the TMA sections was performed using SFRP4 primary antibody, rabbit polyclonal (Protein Tech catalog no: 15328-1-AP) in 1:200 dilution. Heat induced epitope retrieval was performed in TEG buffer (pH 9). Anti-rabbit secondary antibody was used (System- HRP anti Rabbit from Dako Cat No K4003) and visualization done through a chromogen reaction using the DakoEnVision system. Each core on the TMA was scored from 0 to 3 according to the intensity of cytoplasmatic staining of SFRP4. Every core was scored jointly by two physicians and consensus scores were obtained when scoring differed.

### Statistical analysis

All statistical analyses have been performed using STATA version 10. Direct comparisons between clinical groups were done using two-sided t-test statistics. Equal variance assumption has been checked for all t-tests using f-test, and correction for unequal variance has been applied where applicable. Biochemical recurrence status was used as endpoint in multivariate Cox regression analyses. For each variable in the Cox regression analyses, the proportional hazard assumption has been checked using log-log survival curves. Clinical variables included in the multivariate analysis were Gleason score in the prostatectomy specimen, tumor stage and pre-operative PSA level. Gene expression levels were included as a continuous variable. Gleason score was grouped in three groups, high containing Gleason score 8–10, intermediate with Gleason score 7 and low with Gleason score 5–6. Tumor stage was dichotomized in extra prostatic and localized disease. PSA levels were categorized in 3 level groups, low 0–10 ng/ml, intermediate 10–20 ng/ml and high 20+ ng/ml.

## Additional Information

**How to cite this article**: Mortensen, M. M. *et al.* Expression profiling of prostate cancer tissue delineates genes associated with recurrence after prostatectomy. *Sci. Rep.* **5**, 16018; doi: 10.1038/srep16018 (2015).

## Supplementary Material

Supplementary Information

## Figures and Tables

**Table 1:** Clinical and histopathological variables of the study cohort and the validation cohorts.

Clinical Variable	Study cohort	TMA validation cohort	Nakagawa *et al*.	Taylor *et al*.
Age median(range) Years	63 (46–71)	63 (34–76)	66 (47–79)	58 (37–83)
Gleason grade				
Low (5–6)	17 (47%)	154 (33%)	74 (14%)	41 (32%)
Intermediate (7)	15 (42%)	234 (50%)	259 (48%)	74 (57%)
High (8–10)	4 (11%)	82 (17%)	205 (38%)	15 (11%)
Pathological stage				
T2a–c	19 (53%)	308 (66%)	228 (42%)	85 (65%)
T3a–b	17 (47%)	162 (34%)	239 (45%)	35 (27%)
TxN+	0	0	71 (13%)	11 (8%)
Time to recurrence (range) Years	1.3 (0.1–6.2)	1.6 (0.1–8.2)	1.9 (0.1–10.6)	1.6 (0.1–7.7)
Follow up non-recurrent cases Years	5.5 (2.6–6.7)	3.1 (0.1–8.8)	11.7 (4.7–17.9)	4.2 (0.16–12.4)
Recurrence status				
Yes	22 (61%)	168 (36%)	364 (68%)	27 (21%)
No	14 (39%)	302 (64%)	174 (32%)	104 (79%)
Pre-operative PSA (range)	16.0 (5.3–42.5)	11 (1.5–250)	9.4 (0.8–201)	5.9 (1.15–46.4)
Margin status				
Positive	16 (44%)	174 (32%)	NA	31 (24%)
Negative	20 (56%)	366 (68%)	NA	100 (76%)

**Table 2:** Top ranked probe sets associated with recurrence after prostatectomy.

Probe-set	Gene symbol	P-value (Bonferroni corrected)	Fold change (95% CI)
204926_at	INHBA	0.00053	6.2 (3.0–10.5)
234228_at	–	0.0039	5.5 (2.5–9.9)
232473_at	PRPF18	0.011	4.7 (2.0–8.5)
233442_at	–	0.015	5.1 (2.1–9.3)
243586_at	–	0.016	2.9 (1.2–5-4)
215057_at	LOC100272228	0.022	2.5 (1.0–4.7)
211466_at	NFIB	0.028	2.7 (1.1–5.0)
1556879_at	–	0.03	4.4 (1.7–8.3)
219463_at	C20orf103	0.042	3.7 (1.4–6.9)
241676_x_at	–	0.047	4.1 (1.5–7.9)
221011_s_at	LBH	0.048	4.0 (1.5–7.8)

**Table 3:** Clinical and histopathological variables in the molecular subgroups stratified by unsupervised hierarchical cluster analysis.

Clinical variable	Sample cluster 1	Sample cluster 2	Sample cluster 3
Number of samples	13	22	15
Recurrence status (p = 0.022; chi^2^)			
Yes	5 (38%)	17 (77%)	0
No	8 (62%)	5 (23%)	1
Normal samples	0	1	13
Gleason (p = 0.35; chi^2^)			
Low (5–6)	8 (62%)	8 (36%)	1
Intermediate (7)	4 (31%)	11 (50%)	0
High (8–10)	1 (7%)	3 (14%)	0
Pathological stage (p = 0.36; chi^2^)			
T2a–c	8 (62%)	10 (45%)	1
T3a–b	5 (38%)	12 (55%)	0
Pre-operative PSA (range) (p = 0.34; T–test)	15.5 (5.3–42)	16.5 (7.9–42.5)	22,2
Margin status (p = 0.17;chi^2^)			
Positive	4 (31%)	12 (55%)	0
Negative	9 (69%)	10 (45%)	1
