# Supplementary material for: Expression profiling of prostate cancer tissue delineates genes associated with recurrence after prostatectomy
Source: Sci Rep. 2015 Nov 2;5:16018. doi: 10.1038/srep16018 (PMC4629186; doi:10.1038/srep16018)
Supplement: Supplementary Information [file srep16018-s1.pdf]

Supplementary Information

Expression profiling of prostate cancer tissue delineates genes associated with recurrence after prostatectomy

Martin Mørck Mortensen, Søren Høyer, Anne-Sophie Lynnerup, Torben Falck Ørntoft, Karina Dalsgaard Sørensen, Michael Borre and Lars Dyrskjøt

Supplementary Table S1: Signatures from the Markert et. al. study applied to our data set using sample cluster as discriminating factor.

Signature	Normalized p-value	FDR
ESC1	**0.034 ***	**0.18**
ESC2	**0.029 ***	**0.20**
iPCS1	0.83	0.72
iPCS2	0.51	0.53
PRC2	Na	na
p53+	Na	na
p53-	Na	na
PTEN-	0.16	0.19
PTEN+	Na	na
MYC+	0.16	0.24
MYC-	0.94	0.92
ERG1	0.19	0.21
ERG2	0.11	0.22
ERG3	0.088	0.16
ERGcons	**0.039 ***	**0.21**
CYT01	0.14	0.21
CYT02	0.13	0.22
RAS	0.36	0.45
Mesenchyme	0.47	0.53
ProNeural	0.59	0.57
Proliferation	**0.034 ***	**0.23**

Supplementary Table S2: List of transcripts within cluster A, ranked by the association with recurrence

Gene symbol	Study data<br>T-test p-value	Taylor et al.<br>T-test p-value	Nakagawa et al.<br>T-test p-value
INHBA	**0.000001**	**3.63E-07**	**0**
LBH	**0.000009**	0.408355	
SPARC	**0.00002**	**0.001695**	**0.030732**
COL1A2	**0.000039**	**0.001645**	**0.000001**
LOC541471	**0.00004**	0.514036	
C20orf103	**0.00006**	**0.000236**	
COL4A1	**0.000065**	0.057123	
COL4A2	**0.000071**	0.069987	
BGN	**0.000072**	**0.000164**	**0.000001**
COL3A1	**0.000093**	**6.09E-06**	
THY1	**0.000131**	**3.74E-06**	
VCAN	**0.000146**	**0.000644**	
MS4A7	**0.000164**	0.216782	
MSR1	**0.000167**	**0.008024**	**0**
ITGBL1	**0.000243**	**7.3E-06**	
COL1A1	**0.000316**	**3.01E-05**	**0.000664**
DIO2	**0.000317**	**0.043638**	**0.012113**
IGFBP3	**0.000322**	**0.03818**	**0**
HOPX	**0.000508**	**0.006459**	
FN1	**0.000523**	**0.016589**	
MEOX1	**0.000584**	0.385862	
SFRP4	**0.000867**	**2.37E-06**	**0.000001**
RERG	**0.001082**	0.162827	
SULF1	**0.00128**	**0.046025**	
ADAMTS8	**0.001663**	0.620575	
CDH11	**0.001788**	**0.014462**	0.258717
LAMA2	**0.001806**	0.166931	
RAB6B	**0.001868**	**0.010159**	
LOC440416	**0.002111**		
CTHRC1	**0.002227**	**4.03E-05**	**0.000003**
COMP	**0.002365**	**1.23E-05**	
ZIC2	**0.002451**	0.23926	
RAB31	**0.002478**	0.242689	
GAS7	**0.002954**	0.45021	0.870332
FBN1	**0.003286**	0.097941	
KIRREL	**0.003329**	**0.043799**	
COL8A1	**0.003345**	**0.000312**	

PLXDC1	**0.003483**	0.067297	**0.000426**
RASSF2	**0.003642**	0.761074	
FAP	**0.003725**	**7.69E-06**	
HMCN1	**0.00415**	0.117368	
COL5A1	**0.004453**	0.093168	
THBS2	**0.004626**	**0.000198**	**0.006906**
SFRP2	**0.004835**	**0.032986**	
GGT5	**0.005632**	**0.028444**	
MICAL2	**0.00654**	0.736095	
SNX22	**0.007996**	0.789602	
NOX4	**0.008024**	**3.18E-05**	**0.000002**
DACT1	**0.008053**	0.616316	
LEF1	**0.008148**	0.498601	
TMEM200A	**0.009268**	**0.00033**	
POSTN	**0.009746**	**1.38E-05**	**0.000003**
HEYL	**0.011117**	**0.003573**	
CXCL14	**0.012025**	**0.001753**	**0.001921**
FOXD1	**0.015669**	0.41899	
GALNTL1	**0.016505**	0.106496	
THBS4	**0.017569**	**5.9E-05**	
FIBIN	**0.027197**	**0.000778**	
LOC100132884	**0.030242**	0.159692	
COL10A1	**0.031162**	**3.05E-05**	
CPXM2	**0.031795**	**0.021039**	
EPPK1	**0.032981**	0.453567	**0.016644**
TMEM119	0.050159	0.264966	
GSN	0.070271	0.952036	
LRRN4CL	0.077838	0.727312	
GREM1	0.084101	**0.042984**	
NTM	0.198022	**0.020506**	
MAPRE2	0.213565	0.585741	0.190016
CTSK	0.290208	0.092263	
MOXD1	0.423484	**0.032369**	
CDC14A	0.424131	0.11608	
C4A /// C4B	0.478385		
TMEM178	0.555918	0.919721	

Supplementary Table S3: List of transcripts within cluster B, ranked by the association with recurrence.

	Study data	Taylor et al.	Nakagawa et al.
Gene symbol	T-test p-value	T-test p-value	T-test p-value
SNHG9	**0.003367**	0.338363	
TOP2A	**0.010023**	**0.015354**	**0.000073**
CASC5	**0.010284**	**0.031835**	
CHML	**0.012606**	0.640827	
GDPD1	**0.014365**	0.147541	
CDCA5	**0.023625**	0.281137	
TPPP	**0.026419**	0.32723	
EPR1	**0.036939**	0.686632	
BIRC5	**0.038611**	0.097507	**0.000522**
TAF4B	**0.04392**	**0.036984**	
CDKN3	**0.045988**	**0.002591**	**0**
TPX2	**0.046419**	**0.010144**	**0**
MND1	0.053357	0.188878	
NUSAP1	0.059873	0.052986	
KIF4A	0.061677	0.066791	
ANLN	0.080536	**0.029807**	
MMP10	0.100803	0.448672	0.165594
AKR1C3	0.102501	0.422639	
HIST1H3B	0.105069	0.138564	
RRM2	0.12587	**0.012864**	
CENPF	0.141439	**0.00423**	
MELK	0.155978	**0.023348**	
BUB1B	0.16268	**0.047881**	
PTTG1	0.166227	0.254674	
MLF1IP	0.212616	**0.007585**	
BID	0.24508	0.972622	
ECT2	0.353332	0.989987	
CCNB1	0.371625	0.245121	**0**
UGT2B4	0.380292	0.354597	
MKI67	0.380802	**0.019581**	**0.000001**
DLX2	0.433021	0.317113	
AURKA	0.433508	0.550696	**0.000007**
YOD1	0.445649	0.520941	
GRB14	0.503905	0.642291	
FAM111B	0.63689	0.883257	
TOX	0.704441	0.415976	
ANKRD30B	0.746343	0.667919	
FANCI	0.859842	0.443562	

LRRC31	0.872053	0.299788	
CRISP2	0.950511	0.230191	

Supplementary Table S4: List of transcripts within cluster C, ranked by the association with recurrence (* reverse direction of association compared to main study)

	Study data	Taylor et al.	Nakagawa et al.
Gene symbol	T-test p-value	T-test p-value	T-test p-value
LOC100272228	**0.000004**		
NFIB	**0.000005**	**0.000275**	
ABCC4	**0.000015**	0.181894	
KIAA0485	**0.000015**		
MTHFSD	**0.000016**	**0.029013**	
TBL1XR1	**0.000016**	0.150361	
PRPF18	**0.000017**	**0.004946**	
KIAA1267	**0.00003**	**0.005212**	
LOC100131613	**0.00003**		
TJP2	**0.000034**	**2.52E-08**	
PBOV1	**0.000058**	0.195667	0.000457 *
ARL17	**0.000059**	**0.043197**	
SPG7	**0.000068**	**0.017375**	
GTF3A	**0.00008**	0.839595	
LOC100128663	**0.000086**		
BMS1P5	**0.000107**	**0.000412**	
RAD52	**0.000112**	0.373494	0.705339
LOC645030	**0.00012**	0.195539	
LOC202181	**0.000123**	**0.013829**	
IPW	**0.000135**		
PAPD4	**0.000167**	0.082489	
TUBE1	**0.000175**	0.070736	
ACPL2	**0.000222**	0.35599	
C15orf29	**0.000222**	0.248148	
SLC25A27	**0.000227**	0.06322	
CYorf15B	**0.00026**	**0.000494**	
CHD9	**0.000271**	**0.000538**	
EIF3B	**0.000282**	0.281333	
WDR27	**0.000283**	0.14552	
ARHGAP6	**0.000321**	**0.015608**	
POLR2J4	**0.000346**	0.449756	
PAPPA	**0.000388**	0.677971	
ZFHX3	**0.000432**	**2E-05**	
TTC17	**0.000486**	**0.001749**	
TMEM75	**0.000528**	0.742339	
MKLN1	**0.000568**	**0.003807**	
AAK1	**0.00068**	0.374105	0.001783 *

HBS1L	**0.000703**	0.237803	
REV3L	**0.000709**	**0.002141**	
MGEA5	**0.000752**	**0.014114**	
TCP11L2	**0.000761**	0.664569	
LOC729222 /// PPFIBP1	**0.000788**		
KIAA1618	**0.000804**	0.821525	
EHF	**0.000874**	**0.027774**	
SFRS4	**0.000907**	**0.000738**	
TTC6	**0.000927**	0.17911	
SNRPN	**0.0011**	0.181083	
OTUD7B	**0.001274**	0.636997	
METT5D1	**0.001294**	0.386292	
PPP2R2A	**0.001321**	0.455846	
PPM1H	**0.001388**	0.449189	
AGFG2	**0.001457**	0.232177	
MEGF6	**0.001492**	0.533018	
DTWD1	**0.001504**	**0.012216**	
LOC648556	**0.00161**		
CCNC	**0.001689**	0.309635	0.031687 *
AHSA2	**0.00172**	0.094591	
ANGEL2	**0.001748**	0.913103	
SORBS2	**0.001779**	**0.00984**	
NANOG	**0.001791**	0.533926	
ARMC8	**0.001879**	**0.024533**	
AMT	**0.001964**	0.690628	
SUFU	**0.001973**	0.491655	
CP110	**0.002017**	**0.031083**	
ANKRD10	**0.002129**	**0.000565**	
DHX30	**0.00222**	0.273625	
GPR22	**0.002252**	0.121612	
CASD1	**0.002307**	0.093997	
ATP5C1	**0.002494**	0.072513	
FLRT2	**0.002529**	0.149927	
TAF15	**0.002591**	0.358396	
USP34	**0.00263**	0.059648	
PBRM1	**0.002744**	0.232586	
THRAP3	**0.002786**	**0.022388**	
SYMPK	**0.002882**	0.880938	
TRIMP1	**0.002941**	0.280871	
RPAIN	**0.00304**	0.724781	
LOC100190986	**0.003078**		
LPP	**0.003176**	**0.015081**	
RBM39	**0.003346**	**0.012464**	

LOC645513	**0.003461**	**0.049228**	
CD47	**0.003865**	**0.018285**	
ADD1	**0.003894**	**0.041568**	
ARHGAP8	**0.003923**	0.300562	
RNPC3	**0.003954**	**9.97E-05**	
ARL17P1	**0.004007**	0.205923	
CUL4A	**0.004212**	**0.029835**	
RBM6	**0.004228**	0.050466	
GRAMD3	**0.004278**	**0.002036**	
RTN4	**0.004343**	0.128052	
UNQ1887	**0.004345**	0.944494	
TUB	**0.004526**	0.119404	
XKR6	**0.004708**	0.251882	
SP3	**0.004772**	0.35642	
LCMT2	**0.004773**	0.933875	
CBFA2T2	**0.005088**	0.484894	
DNAJC7	**0.005447**	0.419614	
PLGLB1 /// PLGLB2	**0.005495**		
FOXP1	**0.005663**	**0.008099**	
MCM3APAS	**0.005699**	0.842851	
LOC100129436	**0.005704**	**0.002016**	
ABI1	**0.005852**	**0.028947**	
XRN1	**0.005904**	**0.034428**	
ME3	**0.005953**	**0.000168**	
N4BP2L2	**0.006779**	**0.000275**	
GSTM4	**0.006846**	**0.000312**	
UBE3A	**0.007019**	0.195747	
FLJ44606	**0.007117**	0.49798	
LOC151877	**0.007903**		
C22orf26	**0.007948**	0.187713	
HNRNPD	**0.008279**	**0.005933**	
SHROOM1	**0.008301**	0.066967	
LOC339047	**0.008305**	0.050615	
IFRG15 /// TOR1AIP2	**0.008566**		
GOLGA9P	**0.008629**	0.223788	
FLJ38717	**0.00864**	0.248313	
NBPF10 /// NBPF11	**0.008761**		
LOC595101	**0.008946**	**0.003423**	
PDE7A	**0.008961**	0.090199	
C3orf31	**0.009148**	0.163749	
SORL1	**0.009205**	0.502208	
ARID5B	**0.009242**	**0.005784**	
RAPGEF6	**0.009344**	0.623557	

UBE2Z	**0.009609**	0.725116	
ZC3H15	**0.009724**	0.122494	
PDXDC2	**0.010179**		
PACRGL	**0.010263**	0.195868	
SFRS15	**0.010269**	0.185972	
ATF7IP	**0.010358**	**0.023529**	
ODZ2	**0.010504**	0.969699	
FAM82B	**0.01083**	0.7101	
PILRB	**0.011032**	0.610155	
PPP1R12B	**0.011116**	**0.006662**	
LOC100131434	**0.011807**	**0.01433**	
FAM118A	**0.011819**	0.559263	
LOC100128439	**0.011982**		
LOC389634	**0.012227**	0.298469	
ARHGEF7	**0.012513**	**0.000124**	
ANKRD36B	**0.012642**	**0.004292**	
FLJ35934	**0.012881**	0.124617	
KANK1	**0.013133**	0.068377	
SFRS18	**0.013547**	**0.000215**	
FLJ45513	**0.013886**	0.475235	
ZNF214	**0.014287**	0.429953	
HNRNPL	**0.014535**	0.532075	
PTPRF	**0.014601**	0.427741	0.436149
ZDHHC17	**0.014703**	0.323353	
ZNF518A	**0.01604**	**0.000675**	
MTF2	**0.016177**	0.174422	
FAM18B2	**0.016397**	**0.006471**	
ZNF224	**0.016569**	0.097373	
LOC728264	**0.017026**	0.358357	
POLR2J2	**0.017749**	0.139124	
LOC349114	**0.01793**	0.101388	
LOC653110	**0.018059**	0.285867	
C12orf30	**0.018426**	0.182276	
RGPD1 /// RGPD2	**0.018499**		
ZNF440	**0.019107**	0.050115	
EIF4G2	**0.019437**	**0.049887**	
ECHDC2	**0.020102**	**0.000527**	
SDHA	**0.020268**		
CAPN3	**0.020543**	**0.003366**	
SLC15A2	**0.021088**	**7.82E-06**	
COG3	**0.021479**	**0.005943**	
PSEN1	**0.021489**	**0.011951**	
MTSS1	**0.021801**	**0.004894**	

DOCK4	**0.022418**	0.239858	
LOC100272216	**0.022445**		
GLMN	**0.02258**	0.368111	
SLC22A3	**0.024676**	**3.12E-06**	
LOC253039	**0.02516**	0.204096	
GABBR1	**0.025773**	0.884096	
ITPR2	**0.025955**	**0.000216**	
CLIP4	**0.032426**	**0.00042**	
GOLGA8A	**0.032855**		
SCLT1	**0.033588**	0.161685	0.000015 *
AMY1A	**0.035141**		
UBE2D3	**0.03608**	0.061347	
PON2	**0.036465**	0.235051	
LOC100131564	**0.039683**	**0.000106**	
COL4A5	**0.04028**	**0.004853**	
FSTL1	**0.040922**	0.157865	
TPM1	**0.041443**	**3.04E-06**	
SNRNP70	**0.042052**	**0.002339**	
TAF11	**0.043427**	0.536748	
SPTAN1	**0.044919**	0.051281	
TBC1D8	**0.046012**	**0.023378**	
C3orf25	**0.047886**	**0.00894**	
LOC100130097	**0.049967**	**0.009477**	
SLC25A37	0.0517	0.737627	
MKL2	0.052814	**0.003543**	
OXR1	0.05347	0.194192	
KIF5C	0.053476	0.928698	
PANK2	0.054663	0.104665	
TMEM50B	0.054892	0.291704	
PYROXD2	0.056368	0.8694	
TYMS	0.060652	**0.000559**	0.039422 *
NACA	0.063725		
LOC729298	0.065954		
LRRC1	0.071334	0.622411	
PCCA	0.071353	**9.9E-05**	
LOC642776	0.075408	0.714136	
LOC646014	0.080783		
EAF2	0.08114	**0.0003**	
ZNF589	0.081305	**0.002044**	
LOC100131998	0.08156	**0.002814**	
KDM4B	0.081671	0.075806	
PSMG4	0.088607	0.433442	
KLK2	0.090259	0.055647	0.986147

KCNQ1OT1	0.090374	0.644378	
BCL2L11	0.094387	0.912633	
LOC440993	0.096181	0.231514	
TBX3	0.097074	0.053219	
ZNF236	0.09729	0.087746	
RANBP9	0.098638	0.389311	
RAB18	0.101282	0.239031	
CSAD	0.104694	**0.016683**	
AKAP13	0.106356	0.079881	
RUFY3	0.109281	**0.000124**	
PDLIM5	0.117067	**0.000183**	0.000009 *
NKTR	0.119319	**0.010023**	
LAT /// SPNS1	0.119968		
INTS10	0.121392	**0.005329**	
NUMA1	0.125153	0.10097	0.57881
MASP2	0.129493	0.059881	
RBM9	0.140009	**0.013946**	
SH3GLP2	0.145899		
GOLGA8A	0.159269	**0.002237**	
ARL17 /// ARL17P1	0.165487		
TRIM4	0.193614	0.194932	
HEATR7A	0.198273	0.210507	
MGP	0.200689	**0.027734**	
MLLT3	0.20681	0.690357	0.790793
DST	0.210155	**0.000237**	
OGT	0.21456	**0.001143**	
SF1	0.216163	**0.002826**	0.001673 *
RPL10A	0.218948		
KIAA1919	0.27045	0.690477	
C1orf84 /// KIAA0467	0.272012		
STT3B	0.272423	0.370587	
EME2	0.295033	0.149937	
C6orf134	0.30527	0.268633	
LOC100131731	0.324933	0.108804	
FLJ43663	0.334252		
IQCG	0.342303	0.534071	
LOC642852	0.424491	**0.031841**	
PABPC1L	0.44246	0.524071	
MYH11	0.457974	**3.08E-07**	0.028429 *
DKFZP434C153	0.458493		
ZNF334	0.632008	**6.94E-08**	
MUC20	0.90148	0.915591	
NAP1L5	0.974591	0.108393	

Supplementary Table S5: Validation using Gene Set Enrichment Analysis: Gene clusters identified by unsupervised clustering applied as gene signatures to data sets from Taylor et al. and Nakagawa et al.

* The signature is enriched in the non-recurrent group

	Taylor dataset Normalized p-value	Taylor dataset False Discovery Rate	Nakagawa dataset Normalized p-value	Nakagawa dataset False Discovery Rate
Cluster A (Invasive)	**0.025**	**0.02**	0.089	0.391
Cluster B (Cell cycle)	**0.094**	**0.207**	0.083	0.231
Cluster C (Tumor suppressors)	***0.000**	***0.071**	0.145	0.344

Supplementary Table S6: Transcript validation in the two validation data sets.

	Taylor et. al.			Nakagawa et. al.		
Cluster	A	B	C	A	B	C
Transcripts	62	10	195	18	4	6
Validated in T-test (p<0.05)	36	5	69	16	4	0
Validated in T-test (p-value Bonferroni corrected)	20	1	11	11	4	0
Multivariate Cox regression*	na	na	na	10	2	0

Supplementary Table S7: Single transcripts significantly associated with recurrence in multivariate Cox regression analysis

Gene Symbol	Hazard Ratio (95% CI)	p-value
Cluster A (Invasive)		
INHBA	1.48 (1.22-1.81)	<0.001
COL1A2	1.94 (1.32-2.87)	0.001
IGFBP3	1.49 (1.18-1.87)	0.001
MSR1	1.41 (1.10-1.79)	0.006
SFRP4	1.32 (1.07-1.63)	0.008
POSTN	1.26 (1.05-1.52)	0.014
EPPK1	1.23 (1.03-1.48)	0.026
CTHRC1	1.23 (1.02-1.49)	0.03
PLXDC1	1.55 (1.04-2.32)	0.03
NOX4	1.25 (1.00-1.56)	0.048
Cluster B (Cell Cycle)		
TPX2	1.41 (1.13-1.76)	0.002
CDKN3	1.55 (1.16-2.06)	0.003

Supplementary Table S8: Association between cytoplasmatic SFRP4 immunostaining intensity and clinical parameters of the patient cohort

Variable	Categorization	Intensity 0	Intensity 1	Intensity 2	Intensity 3	p-value
Age	Under/equal median	13 (5%)	86 (35%)	115 (47%)	29 (12%)	
	Over median	7 (3%)	89 (39%)	105 (46%)	26 (11%)	0.588
Gleason Grade	5 to 6	6 (4%)	59 (38%)	75 (49%)	14 (9%)	
	7	12 (5%)	86 (37%)	105 (45%)	31 (13%)	
	8 to 10	2 (2%)	30 (37%)	40 (49%)	10 (12%)	0.816
T-stage	T2a-c	17 (6%)	109 (35%)	151 (49%)	31 (10%)	
	T3a-b	3 (2%)	61 (39%)	68 (44%)	24 (15%)	0.085
PSA	under 10	7 (4%)	71 (36%)	89 (46%)	28 (14%)	
	10 to 20	10 (5%)	71 (36%)	97 (49%)	18 (9%)	
	over 20	3 (4%)	33 (42%)	34 (43%)	9 (11%)	0.685
Recurrence	no	13 (4%)	122 (40%)	138 (46%)	29 (10%)	
	yes	7 (4%)	53 (32%)	82 (49%)	26 (15%)	0.128

Supplementary Table S9: Details of the multivariate Cox regression analysis details. The clinical variables are grouped as described in Materials and methods.

	Hazard ratio	p-value	95% confidence interval
SFRP4 expression	1.35	0.009	1.08 - 1.69
T-stage	1.81	0.002	1.25 – 2.63
Gleason grade	1.71	>0.001	1.34 – 2.18
PSA Level	1.64	>0.001	1.32 – 2.05
Margin status	1.97	0.002	1.38 – 2.81
